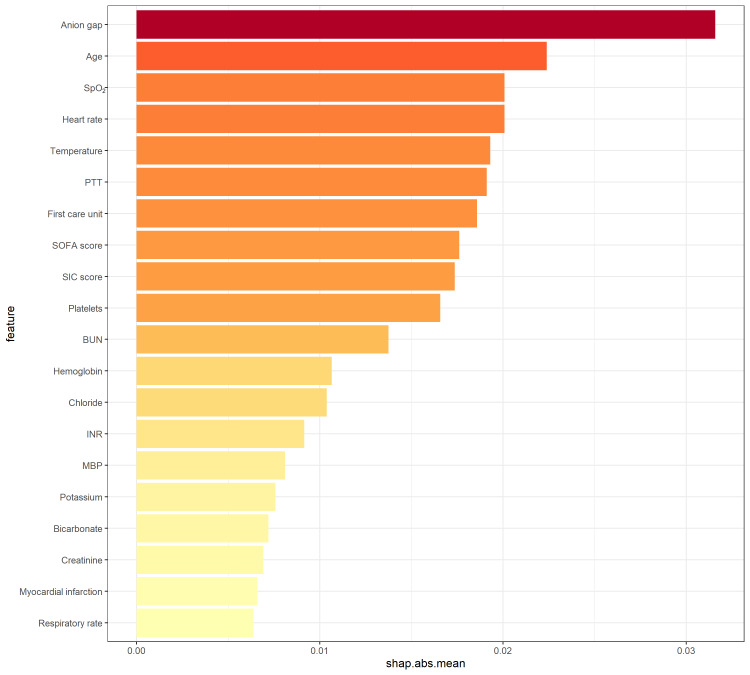# Improving predictions: Enhancing in-hospital mortality forecast for ICU patients with sepsis-induced coagulopathy using a stacking ensemble model

**DOI:** 10.1097/MD.0000000000037634

**Published:** 2024-04-05

**Authors:** Xuhui Liu, Hao Niu, Jiahua Peng

**Affiliations:** a Youjiang Medical University for Nationalities, Baise, China; b Baise People’s Hospital, Baise, China; c Beijing Neurosurgical Institute, Beijing Tiantan Hospital, Capital Medical University, Beijing, China.

## Abstract

The incidence of sepsis-induced coagulopathy (SIC) is high, leading to increased mortality rates and prolonged hospitalization and intensive care unit (ICU) stays. Early identification of SIC patients at risk of in-hospital mortality can improve patient prognosis. The objective of this study is to develop and validate machine learning (ML) models to dynamically predict in-hospital mortality risk in SIC patients. A ML model is established based on the Medical Information Mart for Intensive Care IV (MIMIC-IV) database to predict in-hospital mortality in SIC patients. Utilizing univariate feature selection for feature screening. The optimal model was determined by calculating the area under the curve (AUC) with a 95% confidence interval (CI). The optimal model was interpreted using Shapley Additive Explanation (SHAP) values. Among the 3112 SIC patients included in MIMIC-IV, a total of 757 (25%) patients experienced mortality during their ICU stay. Univariate feature selection helps us to pick out the 20 most critical variables from the original feature. Among the 10 developed machine learning models, the stacking ensemble model exhibited the highest AUC (0.795, 95% CI: 0.763–0.827). Anion gap and age emerged as the most significant features for predicting the mortality risk in SIC. In this study, an ML model was constructed that exhibited excellent performance in predicting in-hospital mortality risk in SIC patients. Specifically, the stacking ensemble model demonstrated superior predictive ability.

## 1. Introduction

Sepsis refers to a clinical syndrome characterized by physiological, pathological, and biochemical abnormalities resulting from a dysregulated response of the organism to infection, leading to life-threatening organ dysfunction.^[1]^ According to the Global Burden of Disease Study Analysis released in 2020, sepsis resulted in approximately 48.9 million cases globally, along with 11.0 million associated mortality cases.^[2]^ Sepsis patients in the early stages can exhibit a complex prothrombotic state characterized by activation of the exogenous coagulation pathway, cytokine-induced coagulation amplification, inhibition of anticoagulant pathways, and fibrinolysis impairment.^[3,4]^ The diagnostic criteria for sepsis-induced coagulopathy (SIC) aim to identify patients in the early stages who exhibit reversible alterations in coagulation status. The diagnostic criteria, formulated in 2017 by the members of the International Society on Thrombosis and Haemostasis Subcommittee on Disseminated Intravascular Coagulation (DIC) Scientific and Standardization Committee, comply with the updated definition of sepsis.^[5]^The SIC score comprises 3 components: platelet count, international normalized ratio (INR), and Sequential Organ Failure Assessment (SOFA) score. As a scoring system, a SIC score ≥ 4 indicates the diagnosis of sepsis-induced coagulopathy.^[6]^ The study revealed that as the SIC score increases, the mortality risk demonstrates a linear escalation, with a mortality rate exceeding 45% at a score of 6.^[5]^

In a study conducted in China, it was found that 67.9% of patients diagnosed with sepsis 3.0 also met the diagnostic criteria for SIC.^[7]^ SIC, as a highly prevalent condition, currently lacks specific therapeutic interventions. It is characterized by a high mortality rate and poor prognosis, making it one of the common causes of mortality in the intensive care unit (ICU).^[8]^ The progression from SIC to DIC is a continuum, with the pathological and physiological characteristics of SIC being characterized by a hypercoagulable state, which then transitions toward a hypocoagulable state during the DIC phase.^[9]^ Hence, early identification of mortality risk in SIC contributes to timely intervention, preventing further immune dysregulation and thereby reducing mortality rates. Given the time-sensitive nature of SIC treatment, early prediction of mortality risk in the early stages of SIC is crucial for improving survival rates in patients with SIC.

Machine learning, as a vital component of artificial intelligence, is a strategy that enables data to speak for itself as much as possible. It can overcome the limitations of traditional clinical statistical methods in interpreting high-dimensional, non-linear, and longitudinal electronic medical record data.^[10]^ In recent years, the clinical application scope of ML has expanded from diagnosis to prediction and has been utilized across various clinical domains. ML algorithms have also been employed for prognostication of critically ill patients.^[11]^ Research indicates that machine learning algorithms can enable early and dynamic prediction of SIC based on medical data.^[12]^ However, there is still a lack of effective tools in clinical practice for predicting the mortality risk of SIC. The aim of this study is to establish and validate a ML model for predicting in-hospital mortality rates in patients with SIC.

## 2. Methods

### 2.1. Data source

Extract data of SIC patients from the Medical Information Mart for Intensive Care IV (MIMIC-IV) open clinical database for the purpose of developing machine learning models. The MIMIC-IV database systematically collected data from sepsis patients in the ICUs of Beth Israel Deaconess Medical Center in Boston, Massachusetts, USA, from 2008 to 2019. This project has received approval from the Institutional Review Boards of the Massachusetts Institute of Technology and Beth Israel Deaconess Medical Center. In order to apply for access to this database, we have successfully completed an examination on the protection of human research participants and obtained a certificate (Certificate Number: 50618389). All health data of patients in this database have been de-identified, thereby obviating the need for obtaining informed consent from the patients. This study was conducted in accordance with the principles of the 2013 Helsinki Declaration.

### 2.2. Study population

Following are the inclusion criteria: Age ≥ 18 years; Initial hospitalization and first admission to the ICU; ICU length of stay > 1 day; Conforming to the diagnostic criteria of Sepsis 3.0, wherein sepsis is defined as a suspected infection accompanied by a rapid increase in SOFA score ≥ 2^1^; SIC score ≥ 4 (Supplementary Table 1, http://links.lww.com/MD/L995), based on the worst daily values of SIC-related indicators during ICU hospitalization. The Conditions for exclusion are as follows: Patients with ≥ 2 admissions to the ICU; Data missing ≥ 20%.

### 2.3. Data collection and results

The variables collected in this study are based on 7 aspects: Demographic characteristics: age, gender; First care unit; Vital signs on the first day of ICU admission: heart rate, respiratory rate, mean blood pressure (MBP), body temperature, arterial oxygen saturation (SpO_2_); Scoring scales on the first day of ICU admission: SOFA score; SIC score; Laboratory test results: hematocrit, hemoglobin, platelets, white blood cell, anion gap, bicarbonate, blood urea nitrogen (BUN), calcium, glucose, chloride, creatinine, sodium, potassium, absolute (Abs) basophils, Abs eosinophils, Abs lymphocytes, Abs monocytes, Abs neutrophils, INR, prothrombin time (PT), partial thromboplastin time; Complications: myocardial infarction, congestive heart failure, chronic pulmonary disease, diabetes, hypertension; Length of hospital ICU stay. We utilized laboratory test results and blood biomarker levels measured within the first day of ICU hospitalization. In cases where multiple measurements were taken within the first day, we used the minimum value for the respective indicators.

The outcome event for this study is the in-hospital mortality rate of SIC patients.

### 2.4. Data preprocessing

We will employ multiple imputation to fill missing values for variables with < 20% missingness (Supplementary Table 2, http://links.lww.com/MD/L996).

### 2.5. Statistical analysis

The distribution of continuous variables will be assessed using the Kolmogorov-Smirnov test. Parametric continuous variables will be assessed using t-tests and expressed as mean and standard deviation. Non-parametric continuous variables will be assessed using the Mann–Whitney U test and expressed as median with interquartile range. Categorical variables will be presented as numbers (percentages) and assessed using the χ² test or Fisher exact test. All statistical tests will be conducted as 2-tailed tests. SPSS software will be used for data computation and statistical analysis. The ML models and Receiver Operating Characteristic (ROC) curves were generated using R software (version 4.3.0). *P* < .05 indicates statistical significance.

### 2.6. Feature engineering technique based on univariate feature selection

We used univariate feature selection technique to choose the optimal subset of predictive features. Univariate feature selection aims to reduce feature dimensionality and enhance model performance. This technique independently assesses the statistical relationships between each feature and the target variable, selecting features that provide the most information value for predicting the target variable.^[13]^ We utilized the Support Vector Machine with the Gaussian kernel for modeling. Robustness and generalization of the model were ensured through 5-fold repeated cross-validation, and detailed training results were saved. Furthermore, the model parameters were tuned through 3 repetitions of cross-validation, utilizing ROC as the evaluation metric. We also enabled the functionality for class probability computation and probability prediction of the model.

### 2.7. Model development and validation

We selected Extreme Gradient Boosting (XGBoost),^[14]^ Random Forest (RF),^[15]^ k-Nearest Neighbors,^[16]^ support vector machine,^[17]^ Light Gradient Boosting Machine,^[18]^ Decision Tree, Logistic Regression,^[19]^ Elastic Net,^[20]^ Single Hidden Layer Neural Network (SHLNN),^[21]^ and a Stacking Ensemble Model (Elastic Net + SHLNN + XGBoost)^[22]^ to construct the mortality risk prediction model. For this purpose, we performed a 10-fold cross-validation process on the input data. The performance of the ML models was assessed by calculating the area under the curve with a 95% CI, as well as accuracy, precision, recall, and F1 Score. By comparing the area under the curve (AUC) of each model, we determined the optimal model. We also conducted decision curve analysis and calibration curve analysis. The use of the Shapley Additive Explanation (SHAP)^[23]^ algorithm provided interpretability for the optimal model, quantifying the contribution of each feature to the predictions made by the best model, while also analyzing 2 case studies.

## 3. Results

### 3.1. Baseline characteristics and feature selection

**Figure 1.**
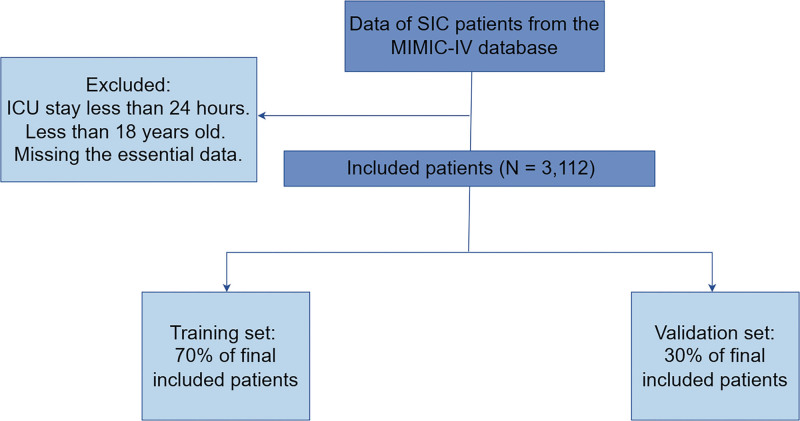
Case screening process flowchart. ICU = intensive care unit, MIMIC-IV = medical information mart for intensive care IV, SIC = sepsis-induced coagulopathy.

Among the 14,804 sepsis patients recruited from the MIMIC-IV database, 3112 were diagnosed with SIC. A total of 757 patients experienced mortality during hospitalization, while 2355 patients did not. The case screening process is illustrated in Figure 1. The characteristics of SIC patients are presented in Table 1.

**Table 1:** Baseline characteristics between the survival group and death groups in the MIMIC-IV cohort.

Items	Survival group (n = 2355)	Death group (n = 757)	Statistic	*P*
Male, n (%)	1493 (47.98)	471 (15.13)		.559
Age (yr), M (Q1, Q3)	65.00 (53.00,77.00)	66.00 (56.00,80.00)	Z = 2.89	.004
SOFA score, M (Q1, Q3)	4.00 (3.00,6.00)	5.00 (3.00,7.00)	Z = 10.53	<.001
SIC score, mean (SD)	5.01 (0.71)	5.38 (0.67)		<.001
First care unit, n (%)				<.001
CCU	159 (5.11)	65 (2.09)		
CVICU	417 (13.40)	27 (0.87)		
MICU	754 (24.23)	301 (9.67)		
MICU/SICU	610 (19.60)	206 (6.62)		
Neuro intermediate	5 (0.16)	1 (0.03)		
Neuro SICU	20 (0.64)	13 (0.42)		
Neuro stepdown	1 (0.03)	2 (0.06)		
SICU	244 (7.84)	77 (2.47)		
TSICU	145 (4.67)	65 (2.09)		
Vital signs				
Heart rate (beats/min), mean (SD)	74.47 (16.04)	78.51 (17.81)	t = 5.56	<.001
MBP (mm Hg), mean (SD)	74.47 (16.04)	51.83 (14.68)	t = −6.60	<.001
Respiratory rate (beats/min), mean (SD)	12.56 (3.83)	13.47 (4.58)	t = 4.93	<.001
Temperature (°C), M (Q1, Q3)	36.40 (35.90,36.70)	36.30 (35.60,36.60)	Z = −6.11	<.001
SpO_2_ (%), M (Q1, Q3)	93.00 (90.00,95.00)	91.00 (88.00,94.00)	Z = −8.88	<.001
Laboratory tests				
Hematocrit (%), mean (SD)	27.84 (6.39)	26.94 (6.54)	t = −3.35	<.001
Hemoglobin (g/dL), mean (SD)	9.26 (2.14)	8.84 (2.15)	t = −4.69	<.001
Platelets (K/µL), M (Q1, Q3)	99.00 (65.00, 122.00)	74.00 (43.00, 108.00)	Z = −9.53	<.001
WBC (K/µL), M (Q1, Q3)	8.40 (5.20, 12.30)	8.70 (4.90, 13.50)	Z = 1.10	.271
Anion gap (mEq/L), M (Q1, Q3)	12.00 (10.00, 15.00)	15.00 (12.00, 19.00)	Z = 14.40	<.001
Bicarbonate (mmol/L), mean (SD)	20.00 (4.80)	17.31 (5.77)	t = −11.61	<.001
BUN (mg/dL), M (Q1, Q3)	21.00 (14.00, 35.00)	32.00 (20.00, 54.00)	Z = 12.45	<.001
Calcium (mg/dL), M (Q1, Q3)	7.71 (0.88)	7.64 (1.07)	t = −1.66	<.001
Chloride (mEq/L), mean (SD)	102.04 (7.21)	99.70 (8.34)	t = −6.95	<.001
Creatinine (μmol/L), M (Q1, Q3)	1.00 (0.70, 1.60)	1.50 (1.00, 2.40)	Z = 12.01	<.001
Glucose, mean (SD)	106.10 (35.54)	103.01 (42.16)	t = −1.82	.07
Sodium (mEq/L), mean (SD)	136.02 (5.69)	135.13 (6.99)	t = −3.19	<.001
Potassium (mEq/L), mean (SD)	3.80 (0.60)	3.92 (0.77)	t = 3.95	<.001
Abs basophils (K/uL), M (Q1, Q3)	0.00 (0.00, 1.50)	0.00 (0.00, 0.10)	Z = −7.48	<.001
Abs eosinophils (K/µL), M (Q1, Q3)	0.10 (0.00, 3.70)	0.00 (0.00, 1.10)	Z = −7.22	<.001
Abs lymphocytes (K/µL), M (Q1, Q3)	24.30 (0.90, 91.10)	5.60 (0.60, 71.40)	Z = −4.66	<.001
Abs monocytes (K/µL), M (Q1, Q3)	9.90 (0.50, 38.60)	1.80 (0.40, 39.00)	Z = −2.04	.042
Abs neutrophils (K/µL), M (Q1, Q3)	241.50 (9.30, 814.20)	31.40 (8.40, 821.40)	Z = −1.67	.095
INR, M (Q1, Q3)	1.50 (1.30, 1.70)	1.70 (1.40, 2.10)	Z = 11.97	<.001
PT (s), M (Q1, Q3)	15.90 (14.60, 18.90)	18.30 (15.60, 22.90)	Z = 11.74	<.001
PTT (s), M (Q1, Q3)	31.40 (27.90, 36.70)	34.90 (29.85, 43.65)	Z = 10.37	<.001
Comorbidities, n (%)				
Myocardial infarction	311 (9.99)	141 (4.53)		<.001
Congestive heart failure	734 (23.59)	230 (7.39)		.685
Chronic pulmonary disease	545 (17.51)	188 (6.04)		.340
Diabetes	651 (20.92)	205 (6.59)		.763
Hypertension	890 (28.60)	251 (8.07)		.021
LOS (d), M (Q1, Q3)	3.00 (1.80, 5.90)	4.20 (2.20, 8.45)	Z = 6.50	<.001

Abs = absolute, BUN = blood urea nitrogen, INR = international normalized ratio, LOS = length of hospital ICU stay, M = median, MBP = mean blood pressure, PT = prothrombin time, PTT = partial thromboplastin time, SD = standard deviation, SIC = sepsis-induced coagulopathy, SOFA = sequential organ failure assessment, SpO_2_ = arterial oxygen saturation, WBC = white blood cell.

We utilized the univariate feature selection method to curate a subset of 20 features from the initial pool of 37 for the purpose of model development (Supplementary Figure 1, http://links.lww.com/MD/L997). The selected features encompassed variables such as SOFA score, SIC score, age, hemoglobin, platelets, anion gap, bicarbonate, chloride, potassium, creatinine, BUN, INR, partial thromboplastin time, heart rate, MBP, respiratory rate, temperature, SpO_2_, myocardial infarction, and first care unit.

### 3.2. Comparison of 10 models

**Figure 2.**
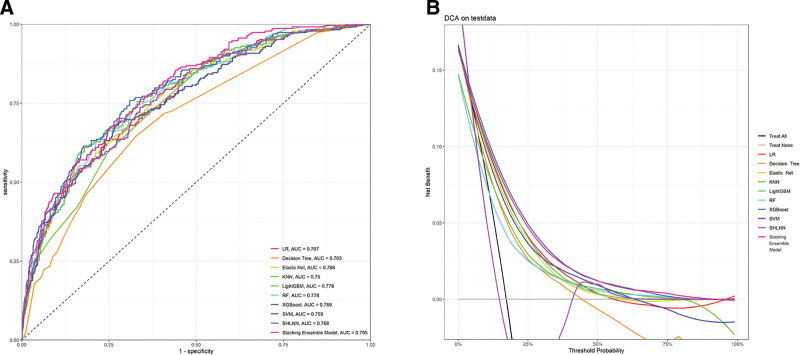
(A) The discriminative ability of the 10 models was compared using ROC curves and AUC. (B) The performance of 10 machine learning models was evaluated using DCA across different decision thresholds. AUC = area under the curve, DCA = decision curve analysis, KNN = k-Nearest neighbors, Light GBM = light gradient boosting machine, LR = logistic regression, RF = random forest, SHLNN = single hidden layer neural network, SVM = support vector machine, XGBoost = extreme gradient boosting.

We ultimately divided the study population into a training set consisting of 2177 individuals (70%) and a test set consisting of 935 individuals (30%). The stacking ensemble model achieved the highest AUC in the testing set (0.795, 95% CI: 0.763–0.827), surpassing individual models such as Elastic Net (AUC: 0.768), SHLNN (AUC: 0.768), and XGBoost (AUC: 0.789) (Fig. 2A). To gain a deeper insight into the performance of these 10 models, we also measured their accuracy, precision, recall, and F1 Score, with the results listed in Table 2. The decision curve analysis curves and calibration curve demonstrate that the stacking ensemble model exhibits the most satisfactory predictability (Fig. 2B, Supplementary Figure 2, http://links.lww.com/MD/L998,).

**Table 2:** Performance comparison of models on the training dataset.

Model	Accuracy	Precision	Recall	F1 Score	AUC-ROC (95% CI)
Decision Tree	0.666	0.389	0.645	0.485	0.703 (0.666–0.741)
KNN	0.657	0.389	0.715	0.504	0.750 (0.715–0.784)
Elastic Net	0.645	0.386	0.772	0.515	0.768 (0.733–0.802)
LR	0.656	0.390	0.728	0.508	0.767 (0.732–0.801)
SHLNN	0.682	0.411	0.702	0.519	0.768 (0.733–0.803)
SVM	0.675	0.405	0.706	0.514	0.759 (0.723–0.795)
Light GBM	0.672	0.408	0.768	0.533	0.778 (0.744–0.812)
RF	0.718	0.447	0.662	0.534	0.778 (0.745–0.814)
XGBoost	0.709	0.442	0.732	0.551	0.789 (0.756–0.823)
Stacking Ensemble Model	0.728	0.461	0.667	0.545	0.795 (0.763–0.827)

95% CI = 95% confidence interval, AUC-ROC = area under the receiver operating characteristic curve, KNN = k-nearest neighbors, Light GBM = light gradient boosting machine, LR = logistic regression, RF = random forest, SHLNN = single hidden layer neural network, SVM = support vector machine, XGBoost = extreme gradient boosting.

### 3.3. Interpretability of the model

**Figure 3.**
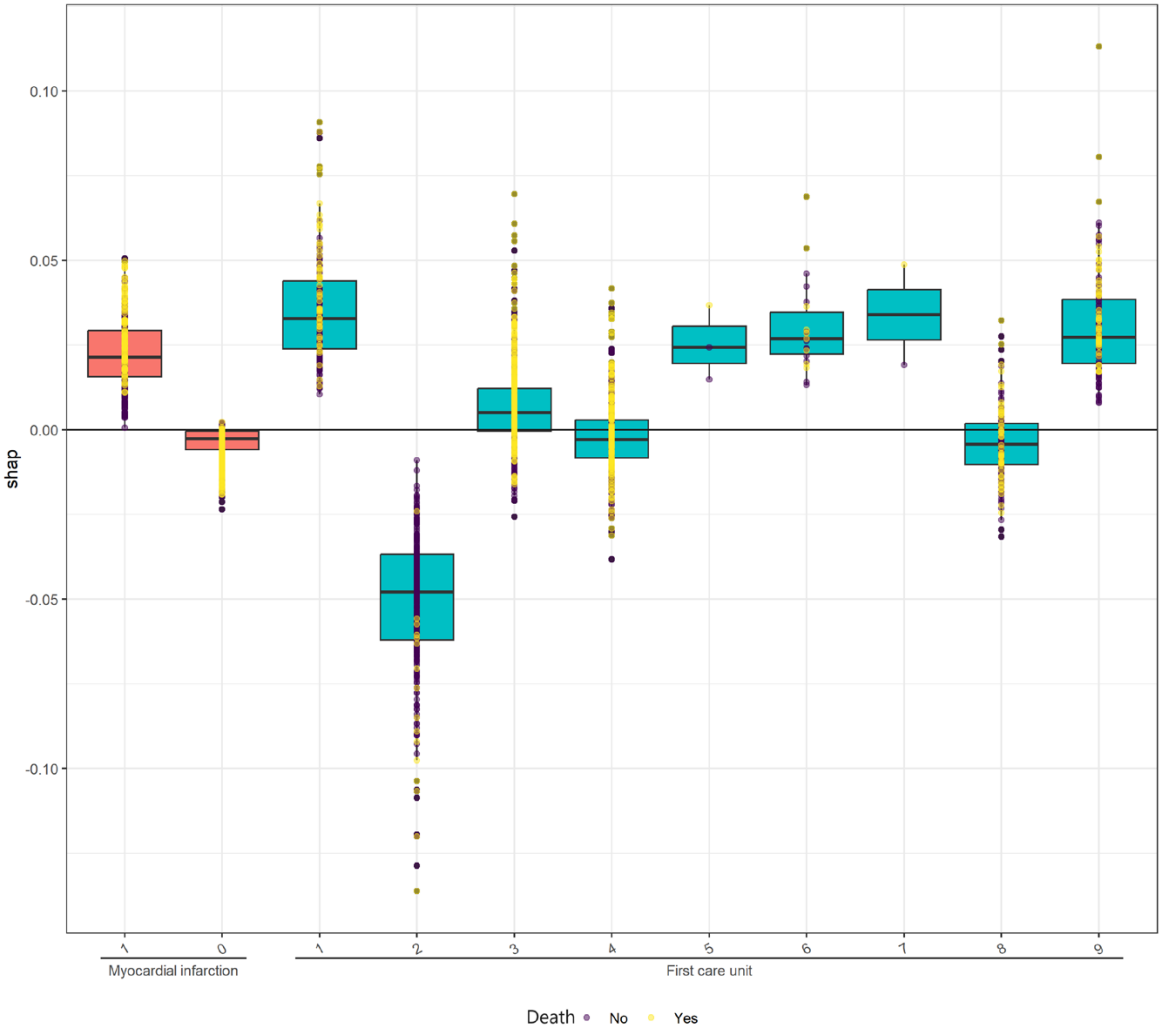
Feature importance of categorical variables. The yellow dots represent deceased samples, while the purple dots represent surviving samples. The horizontal axis represents the names of categorical variables, where 1 indicates presence, and 0 indicates absence. For “First care unit,” 1 to 9 correspond to CCU, CVICU, MICU, MICU/SICU, Neuro intermediate, Neuro SICU, Neuro stepdown, SICU, and TSICU, respectively.

**Figure 4.**
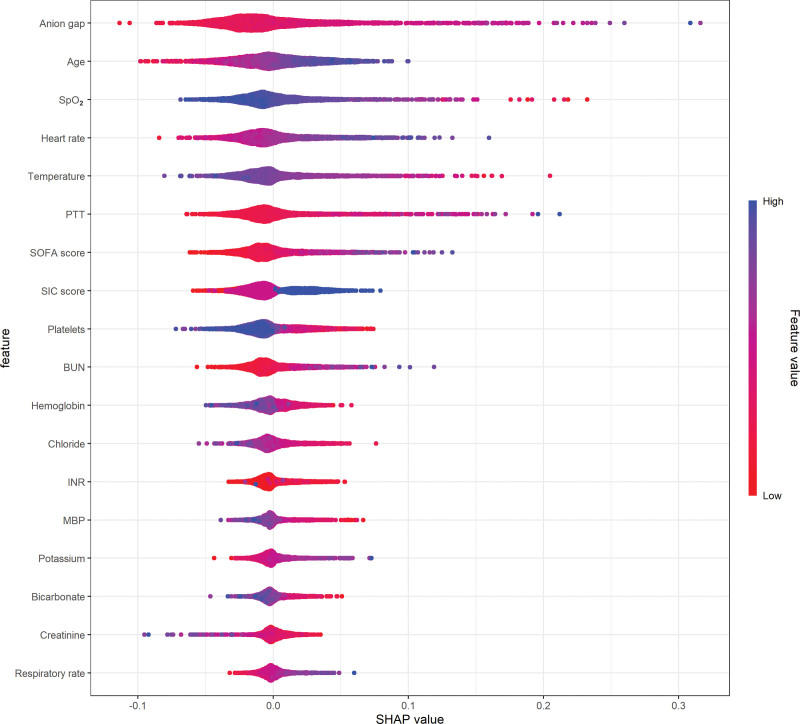
Hive plot. Continuous variables were ranked based on the sum of SHAP values across all patients, with SHAP values used to illustrate the distribution of the impact of each continuous variable on the output of the stacking ensemble model. SpO_2_ = arterial oxygen saturation, BUN = blood urea nitrogen, INR = international normalized ratio, MBP = mean blood pressure, PTT = partial thromboplastin time, SIC = sepsis-induced coagulopathy, SOFA = sequential organ failure assessment.

To gain a deeper understanding of how the ensemble model predicts mortality, we employed the SHAP algorithm to explain the model outcomes. The feature importance of the stacking ensemble model is depicted in the figure (Supplementary Figure 3, http://links.lww.com/MD/L999,). Among the 20 explanatory variables, anion gap was identified as the most important variable, followed by age, SpO_2_, and heart rate. We used SHAP summary plots to illustrate the overall positive and negative impacts of continuous and categorical variables on the output of the stacking ensemble model. Among the categorical variables, first care unit contributes the most to the model value (Fig. 3). Among the continuous variables, anion gap contributes the most to the model value (Fig. 4).

**Figure 5.**
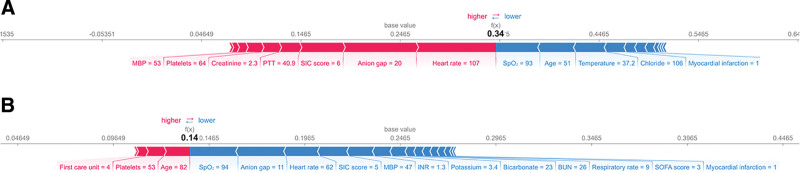
The force plot illustrates interpretable examples of single-sample feature prediction results. Red indicates a positive impact on the model outcome, while blue denotes a negative impact. The length represents the importance of features. The baseline value (0.25) represents the average of the prediction model; the output value represents the predicted risk of death. The figure provides an interpretation of the death instance (A) and the survival instance (B). SpO_2_ = arterial oxygen saturation, BUN = blood urea nitrogen, INR = international normalized ratio, MBP = mean blood pressure, PTT = partial thromboplastin time, SIC = sepsis-induced coagulopathy, SOFA = sequential organ failure assessment.

**Figure 6.**
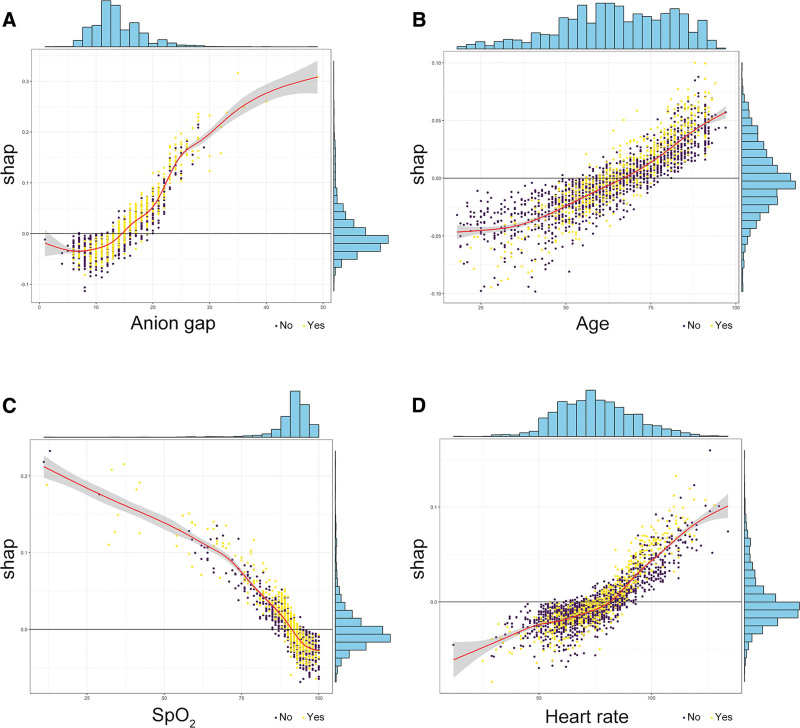
Univariate SHAP plots. Yellow represents deceased samples, while purple represents surviving samples. (A) Displays the positive impact of high anion gap values on the model prediction results. (B) Illustrates the positive impact of advanced age on prediction outcomes. (C) Indicates the negative influence of high SpO_2_ values on the prediction results. (D) Demonstrates the positive impact of increased heart rate on the model prediction results.

We randomly selected 2 samples and employed the SHAP analysis method to interpret the predictive results of the stacking ensemble model. The red and blue bars represent positive and negative effects, respectively; longer bars indicate higher functional importance. We conducted an interpretation using instance number 979, where heart rate = 107 and anion gap = 20 played a predominant positive role in predicting the outcome, while SpO_2_ = 93 and age = 51 had a significant negative impact. The model output value was 0.34, surpassing the baseline of 0.25, and successfully predicted the patient as an in-hospital mortality case (Fig. 5A). For instance number 1174, age = 82 and platelets = 53 had a noteworthy positive impact on predicting the outcome. The final model output value was 0.14, which was lower than the baseline of 0.25, and the model successfully predicted the patient as a survivor (Fig. 5B). Figure 6 summarizes the univariate distributions of the top 4 continuous variables in the stacking ensemble model output.

### 3.4. Web development

We will deploy the stacking ensemble model we have constructed on a Shiny web page, making it easily accessible for clinical practitioners. The website can be accessed at the following link: https://saexgboost.shinyapps.io/SIC1/. Using this website, one can assess the mortality risk of SIC and display the predictive results to the users.

## 4. Discussion

Machine learning has found widespread applications in the medical field, revolutionizing healthcare by enhancing the efficiency and accuracy of diagnosis, treatment, and patient care.^[24]^ In this article, we explore the effectiveness of machine learning in predicting the mortality risk of SIC patients and provide explanations for the best-performing model.

Our study demonstrates that the stacking ensemble model can predict in-hospital mortality risk in SIC more accurately than other algorithms. The ensemble model algorithm employs a Stacking approach,^[25]^ combining 3 base models, namely XGBoost, Elastic Net, and SHLNN (referred to as primary learners), to create a meta-model (also known as a secondary learner), thereby enhancing the model performance. It has 2 main advantages. Firstly, it combines the 3 base models in a flexible manner, leveraging the differences between these models to enhance the predictive performance of the overall model. Secondly, by employing a meta-model, Stacking is capable of amalgamating the predictions generated by the 3 base models. This fusion approach can reduce model bias, enhance model robustness, and improve generalization capabilities.

In this research, univariate feature selection was employed to choose 20 variables for the construction of a machine learning model. These variables are common indicators easily obtainable in clinical practice. Additionally, we have developed a Shiny web tool to facilitate the convenient utilization of this integrated model by clinicians for predictive purposes, thereby providing a useful reference for further clinical decision-making.

In this study, anion gap emerged as the most crucial variable in the clinical mortality prediction model for SIC, followed by vital signs (temperature and SpO_2_). Renal function parameters such as BUN, SpO_2_, and potassium contribute to the assessment of SIC risk. Several studies have also indicated a close association between the above-mentioned indicators and the risk of mortality in SIC patients. Numerous studies have validated the association between serum anion gap and the mortality rate of critically ill patients,^[26–28]^ with the anion gap serving as a risk factor for long-term extracorporeal support.^[29]^ We have ample evidence to support the notion that the anion gap can serve as a predictive factor for forecasting the mortality rate among SIC patients. A retrospective single-center cohort study by Erez Marcusohn et al indicated an association between a body temperature > 39.5°C and adverse clinical course.^[30]^ Serum creatinine and BUN are important indicators for evaluating renal function. Measuring these 2 parameters in the early stages of SIC patients can aid in assessing renal function, identifying sepsis-related acute kidney injury, and predicting disease progression and prognosis.^[31]^ As an essential physiological parameter in the human body, SpO_2_ serves to assess the circulatory system functionality and stands as a crucial monitoring indicator in early resuscitation protocols.^[32]^ Lara Hessels et al’s study revealed a close association between potassium disturbances and in-hospital mortality, which persisted even after adjusting for disease severity and acute kidney injury (AKI).^[33]^ However, there is limited research available regarding the relationship between serum potassium levels and mortality in SIC patients.

To the best of our knowledge, this study represents the inaugural attempt at applying a stacking ensemble model to predict the mortality risk of patients with SIC. In comparison to extant solutions, our research introduces an innovative stacking ensemble model approach that successfully forecasts in-hospital mortality risk among SIC patients in the ICU, thereby offering a valuable supplement to current clinical decision-making methods. The performance of our stacking ensemble model in mortality prediction significantly surpasses that of traditional machine learning methods, demonstrating its efficacy in discerning crucial clinical features among SIC patients. However, it is imperative to acknowledge certain limitations in this study. Despite the partitioning of the dataset into training and validation sets, the challenge of insufficient multicenter data for a more comprehensive model validation persists. Compared to recent advancements in sepsis management, the management of sepsis-induced coagulopathy still has a long way to go.

## 5. Conclusion

This study demonstrates the effectiveness of the stacking ensemble model in predicting in-hospital mortality risk among SIC patients. The anion gap plays a role in the in-hospital mortality risk of SIC patients.

## Author contributions

**Data curation:** Hao Niu.

**Writing – original draft:** Xuhui Liu.

**Writing – review & editing:** Jiahua Peng.

## Supplementary Material





**Figure:**
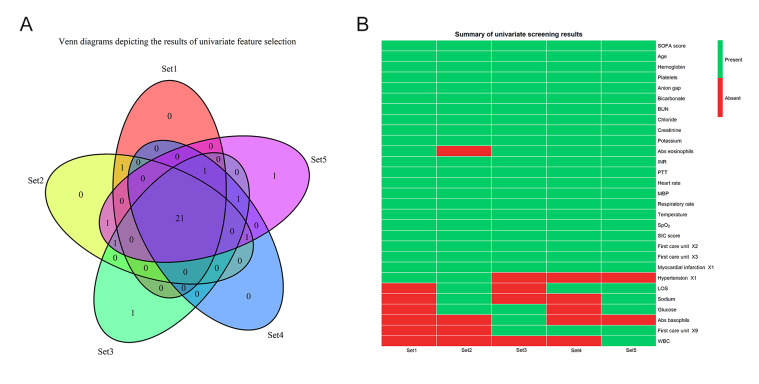


**Figure:**
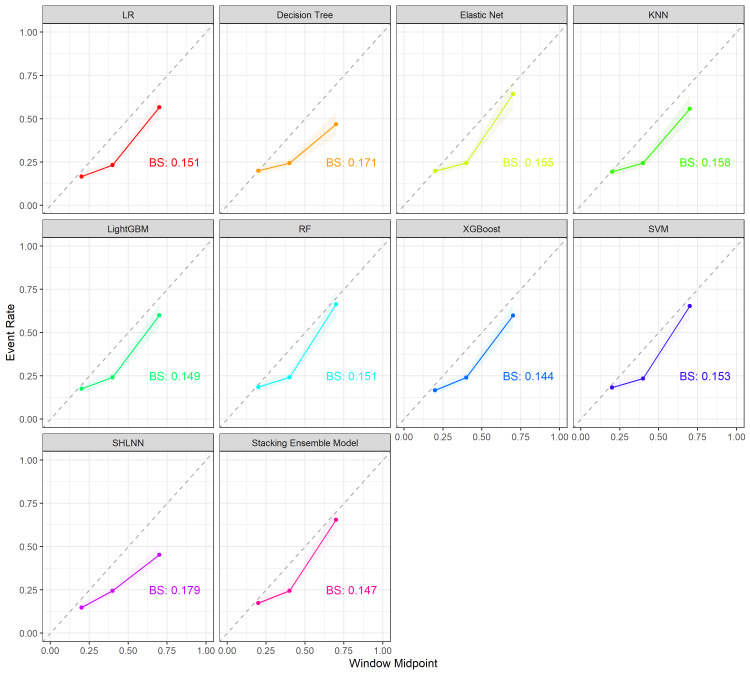


**Figure:**