# Supplementary material for: Improving predictions: Enhancing in-hospital mortality forecast for ICU patients with sepsis-induced coagulopathy using a stacking ensemble model
Source: Medicine (Baltimore). 2024 Apr 5;103(14):e37634. doi: 10.1097/MD.0000000000037634 (PMC10994494; doi:10.1097/MD.0000000000037634)
Supplement: Supplementary file 1 [file medi-103-e37634-s001.docx]

Supplementary Table 1. Sepsis‐induced coagulopathy criteria

|  | Score | Range |
| --- | --- | --- |
| Platelet count (10^3^/μL) | 2 | < 100 |
|  | 1 | ≧ 100, < 150 |
| INR | 2 | > 1.4 |
|  | 1 | > 1.2, ≦1.4 |
| SOFA score | 2 | ≧ 2 |
|  | 1 | 1 |
| Total score for SIC |  | ≧ 4 |

INR = International normalized ratio, SOFA = Sequential organ failure assessment, SIC = Sepsis‐induced coagulopathy.
