# Supplementary material for: Improving predictions: Enhancing in-hospital mortality forecast for ICU patients with sepsis-induced coagulopathy using a stacking ensemble model
Source: Medicine (Baltimore). 2024 Apr 5;103(14):e37634. doi: 10.1097/MD.0000000000037634 (PMC10994494; doi:10.1097/MD.0000000000037634)
Supplement: Supplementary file 2 [file medi-103-e37634-s002.docx]

Supplementary Table 2. Missing number for risk variables and outcome variables

| Risk variables | Missing number (%) |
| --- | --- |
| Age | 0 (0) |
| Gender | 0 (0) |
| SOFA score | 0 (0) |
| SIC score | 0 (0) |
| First care unit | 0 (0) |
| Heart rate | 16 (0.1%) |
| MBP | 16 (0.1%) |
| Respiratory rate | 21 (0.1%) |
| Temperature | 536 (3.6%) |
| SpO_2_ | 19 (0.1%) |
| Hematocrit | 0 (0) |
| Hemoglobin | 187 (10.3%) |
| Platelet | 5 (0) |
| WBC | 3(0) |
| Anion gap | 7 (0.0%) |
| Bicarbonate | 0 (0) |
| BUN | 4 (0.0%) |
| Calcium | 794 (5.4%) |
| Chloride | 2 (0.0%) |
| Creatinine | 1 (0.0%) |
| Glucose | 66 (0.4%) |
| Sodium | 2(0.0%) |
| Potassium | 5 (0.0%) |
| Abs basophils | 0 (0) |
| Abs eosinophils | 0 (0) |
| Abs lymphocytes | 0 (0) |
| Abs monocytes | 0 (0) |
| Abs neutrophils | 0 (0) |
| INR | 566 (3.8%) |
| PT | 566(3.8%) |
| PTT | 628 (4.2%) |
| Myocardial infarction | 0 (0) |
| Congestive heart failure | 0 (0) |
| Chronic pulmonary disease | 0 (0) |
| Diabetes | 0 (0) |
| Hypertension | 0 (0) |
| LOS | 0 (0) |

SOFA = Sequential organ failure assessment, SIC = Sepsis‐induced coagulopathy, MBP = Mean blood pressure, SpO_2_ = Arterial oxygen saturation, WBC = White blood cell, BUN = Blood urea nitrogen, Abs = Absolute, INR = International normalized ratio, PT = Prothrombin time, PTT = Partial thromboplastin time, LOS = Length of hospital ICU stay.
